# The life history narrative of clinical nurses with more than 30 years of experience

**DOI:** 10.1186/s12912-022-00871-9

**Published:** 2022-04-20

**Authors:** Bong Ja Shin, Eun Young Park

**Affiliations:** 1 Gachon University Gil Medical Center, Incheon, South Korea; 2 College of Nursing, Gachon University, Incheon, South Korea

**Keywords:** Life history, Narrative, Nurse clinicians, Qualitative research, Working woman

## Abstract

**Background:**

The nurses with long-term careers in clinical settings shows a clear declining trend. Recording the specific period in Korea’s nursing history is also important from a historical perspective. The aim of this study was to analyze the life history narrative of clinical nurses who have been in service for more than 30 years and to explore the strength and structure of their experience that enabled them to retain their long-term careers.

**Methods:**

This study conducted qualitative research with a life history narrative. For data collection, biographical-narrative interview through in-depth personal interviews with six participants. The participants were clinical nurses who had worked at a general hospital for more than 30 years. The interviews were conducted in three sessions per participant, each session lasting 90–180 min. Qualitative thematic analysis was used to analyze the data.

**Results:**

The narrative of their life stories were analyzed by dividing them into “Dimensions,” “Turnings” and “Adaptations”. The dimensions were categorized into individual and career dimensions. Turning points were empirical and environmental conditions that posed a threat to their career retention as nurse clinicians. Adaptations were illustrated individual methods and social interactions. Twenty-four themes were derived from the 94 thematic statements. After the abstract, four comprehensive categories emerged. The core theme for retaining long-term careers, with “Finding value in myself” comprising four themes: “acknowledgement and support from families,” “healthy relationship at work,” “trusting myself’,” and “accumulation of small achievements.”

**Conclusions:**

The life narratives of participants reveal a close connection with and relationship between the changes in the social aspects, the fields of healthcare and nursing, their individual predispositions, family recognition, and organizational support at that time in Korea. The healthy interpersonal relationships in work place are the most important condition in maintaining the long-term work of nurses. The experience of nurses in one era may not be able to represent experiences in another era, so an in-depth study exploring the social context seems to be necessary. There is a need for policies and changes in the field that can keep the lives of professional women working as nurses proudly.

## Introduction

### Background

The number of nurses with long-term careers in clinical settings shows a clear declining trend. According to a survey on the current staffing status of hospital nurses by Korea Hospital Nurses Association in 2018, 45.5% of new nurses resigned within 1 year. Additionally, among the nurses in service, those with work experience of between one and 3 years accounted for the highest proportion, at 22.0% [1]. The average number of years in service was only 7 years and 8 months [1]. Hard and intensive work, a professional environment that requires working in shifts, and an organizational culture comprising a variety of interpersonal relationships, are factors contributing to the challenges in maintaining a long-term nursing career [2]. Further, the culture of frequent turnover and pursuit of individual quality of life and happiness also serve as factors affecting long-term service [3].

From 2017, approximately 49.6% of the total number of licensed nurses were in actual service in hospitals [4]. This indicates nursing staff shortages. With the aggravating problems of nurse turnover and staff shortages, an analysis of the causes and countermeasures for these problems, as well as establishment of strategies for retaining a skilled nurse workforce, have emerged as issues of key importance [5]. The turnover and resignation of nurses increase the workload and stress for their colleagues still in the workforce [6]. This, in turn, compromises organizational stability, and leads to a shortage of experienced nurses, subsequently lowering the standard of nursing professionalism [7]. Although generation-specific changes in values are desirable for a greater number of nurses to be retained, to be able to provide long-term service. This is due to the inherent characteristics of the nursing profession that requires relevant skills and human care [6].

To investigate the phenomenon of skilled nurses’ career retention, exploring the strength of long-term service will be instrumental. It is also useful to conduct an analysis on the causes of turnover and the establishment of strategies for workforce retention. Further, when considering the historic nature of nursing within the same culture, recording the narrative of a nurse’s life history through interviews with long-term career nurses is necessary. Therefore, through the narrative of nurse clinicians with careers of over 30 years in clinical practice, this study aims to explore the experiences that nurse clinicians undergo in the midst of the changing times and social environments. It also explores the strengths that sustained them in retaining their profession as nurses for this duration.

This study explores the inherent structure and core empirical themes of the experiences that enabled nurses to retain their long-term careers. The study’s findings are expected to lay the groundwork and provide basic data for the development of policies and practices that will help improve nurse retention and address nursing staff shortages.

### Objectives

This study aims to analyze the life history narratives of nurse clinicians in the baby boomer generation to explore the strengths and structure of their experiences. This is in regard to their long-term career retention as nurses for over 30 years. This aim was explored using the following research questions: “What is the lived experience of clinical nurses who have worked over 30 years?” and “What was the most important aspect of career retention over 30 years?

## Methods

### Study design

Life history narrative qualitative research design was conducted. Mandelbaum’s life narrative method influenced this study. This method designed to explore life history narratives through three categories: Dimensions, Turnings, and Adaptations [8].

Since the 30-year careers of nurse clinicians are multi-faceted, to develop a proper understanding of their careers, research using life-history narratives was conducted. It explored the entire life cycle, allowing a consideration of the relevant time- and context-specific issues. This method is regarded as being more appropriate than a cross-sectional study, which investigates only a specific period [9].

### Participants

Six nurses, who had been working at a general hospital for 30 years or more at the time of the study participated. As Patton [10] suggested, the sample size was based on the aim of ensuring thematic data saturation. Thematic data saturation is achieved when the data collected is reviewed repeatedly to try and find relevant themes for analysis. When the analysis no longer produces new information that addresses the research question, it results in thematic data saturation. Additionally, convenience sampling was used to select the participants.

All six participants were female, aged between 54 and 60 years. Four were married and had two or more children. Two were single and lived with their parents. Regarding their educational level, three had a master’s degree and three had a Ph.D. Their years of clinical nursing experience ranged from 30 to 36 years.

### Ethical considerations

The study received ethical approval by the Institutional Review Board of Gachon University (1044396–201,706-HR-102-01). We informed participants that participation was voluntary, their identities would remain anonymous, their information would be kept confidential, and they could withdraw from the study at any time. All study participants provided written informed consent, after the purpose and procedures of the study was explained. They were also informed before they gave consent that the interviews would be recorded, and the advantages and disadvantages of participating was discussed. To ensure that the participants were protected, any identifiable information was excluded from the digital file name saved on the main researcher’s computer. Participants were also informed that the data will only be used for research purposes.

### Data collection

Unlike in the case of standardized interviews, the three steps of a biographical-narrative interview [11] were employed in this study. This is a method of spontaneously narrating one’s life history, as experienced by the speaker in his or her daily life. This occurs without the intervention of the researcher through the speaker’s own choices and decisions. The first step was to build trust. The second was to use open-ended questions, and the third was to use semi-structured questions for further clarification of the interviews’ contents.

The interview guide included three stages. Stage 1: Please describe what your professional life was like starting with whatever comes to mind. Could you talk about the single most memorable moment or event regardless of whether it was a good or a bad one? Stage 2: What were your thoughts or emotions when you considered resigning or changing workplaces? Stage 3: After you considered resigning or changing workplaces, have you experienced any changes in your original thoughts? Please explain the process of how you decided to continue in your original job.

The data collection period was from July 2017 to June 2018. Each participant underwent three sessions of the interview, with each session lasting 90–180 min. An unrestricted environment is required to present oral narratives; therefore, each time, the interviews were conducted at a time and place selected by the study participants. Additional interviews were conducted over the phone or in writing. When no more topics could be derived from the analysis, data saturation was assumed to have been achieved. Hence, we stopped collecting data after six participants.

### Data analysis

The data were analyzed manually by two researchers. They discussed and shared their experiences and opinions regarding data collection and analysis. The initial analysis was conducted by the interviewer, following which the other researcher read the transcription and analyzed them again.

The recorded interviews’ contents were transcribed and summarized. Additionally, we undertook a review process, whereby the presence of any distortion or mis-description of the content was reviewed and clarified by the individual participants through e-mails. For the data completed via the review process, Braun & Clarke’s [12] six steps of thematic analysis method were applied. This is a method that focuses on identifying the common themes for the phenomenon of interest. In the present study, we first repeatedly read transcribed materials and highlighted the contents related to the study’s purpose. Second, initial codes were generated and the interview content was sorted via codes. A total of 94 significant thematic statements were extracted. In the third step, the sorted codes were grouped by candidate themes. Finally, 24 themes were derived from the 94 thematic statements. Once these were analyzed, a collection of four comprehensive and abstract categories emerged. In the fourth step we reviewed whether the grouped themes were related to the transcribed content. In the fifth step, themes were extracted from the categories, and defined and named. To review the themes, the researchers cross-checked the themes in relation to both the coded extracts and the full dataset. These themes were defined and named by writing a detailed analysis and identifying the essence of each. A weaving analytic narrative was written and contextualized. Finally, the three frames of reference—Dimensions, Turnings, and Adaptations—proposed by Mandelbaum [8] were applied and, based on this, the data were analyzed again to achieve the study’s purpose. This involved exploring the strengths of retaining a career for over 30 years.

### Ensuring rigor in research

To ensure rigor in this study, we applied the criteria proposed by Sandelowski [13]: reliability, fittingness, auditability, and confirmability. The data of this study revealed the actual experiences of the nurses who worked in a clinical setting for over 30 years. The individual interviews were recorded and transcribed verbatim. Accuracy of the transcriptions was ensured by comparing the recordings with the transcriptions. Citations were identified to illustrate the themes derived from the data. The participants’ narratives were described and interpreted according to these themes. After the accomplishment of these steps, the manuscript was read multiples times, and all the analytical procedures that led to the initial codes, sub-themes, and themes were described clearly. The researchers also tried to remain unbiased throughout the research process.

## Results

The results of the analyses are explained according to the three dimensions of Mandelbaum’s life history narrative analysis and narrative theme analysis. This allowed the researchers to explore the strengths of career retention over 30 years.

### The life history narratives

The dimensions were categorized into individual and career dimensions. For important turning points in their lives, empirical and environmental conditions that posed a threat to their career retention as nurse clinicians were analyzed. Regarding adaptations, their individual methods of adaption and social interactions were analyzed. These served as strengths that helped the individual nurses overcome the threats they encountered (Table 1).

**Table 1:** Nurses’ life history narratives (via Mandelbaum’s [5] framework)

Dimensions	Turnings	Adaptation
**Life as an individual**		
1. Baby boomer grown up as a model student	1. Nursing major selection based on the will of others and my will	1. Motivating myself
A step toward the dream	- A set path of life	- Let us do it right
Become a college student		
2. Dreamer	2. Infinite responsibilities of childrearing	2. Relying on incomplete childcare support
Who realized the dream of becoming a professional		
Employment	- Nobody to lend a helping hand	- Help from family and friends
Identity as a nurse		
3. Superhero	3. Family economic bankruptcy	
Who juggles multiple things pursuing excellence in all		
Mother who must do well	- Partner’s business failure	
Harmony between work and family life		
Pain and pride at the same time		
**Life as a nurse**		
1. Growth from a novice nurse		
Actual clinical setting aroused feeling of fear and anxiety	1. Interpersonal relationships	1. Acknowledgements from nurse managers
Turnings as a nurse	- Incomprehensible unreasonable criticism	- Encouragement and praise
2. Being a proficient nurse	2. Unreasonable organizational system	2. Break a relationship
Leader in nursing care with new medical technology	- Coercive boss	- Move department
Nothing to be afraid of my work	3. Living on a different schedule	3. Compromise with myself
Struggle to position myself	- Three-shift rotation	- Accept the situation
Reborn as an expert nurse	4. Lack of professional awareness for nurses	4. Authority over my work
3. Professionalism	- Prejudice against women and hospital culture	- Great virtue of nursing
Contemplation on the nature of nursing		
Nursing profession under reflection		

In Life as an individual domain, the following were derived: baby boomers growing up as model students, a dreamer who realized the dream of a professional, and a superhero who juggles multiple things pursuing excellence in all domains. Life as a nurse domain was divided into three areas: growth from a novice nurse, being a proficient nurse, and professionalism.

### Narratives of essential strength that enable long-term career retention

**Fig. 1:**
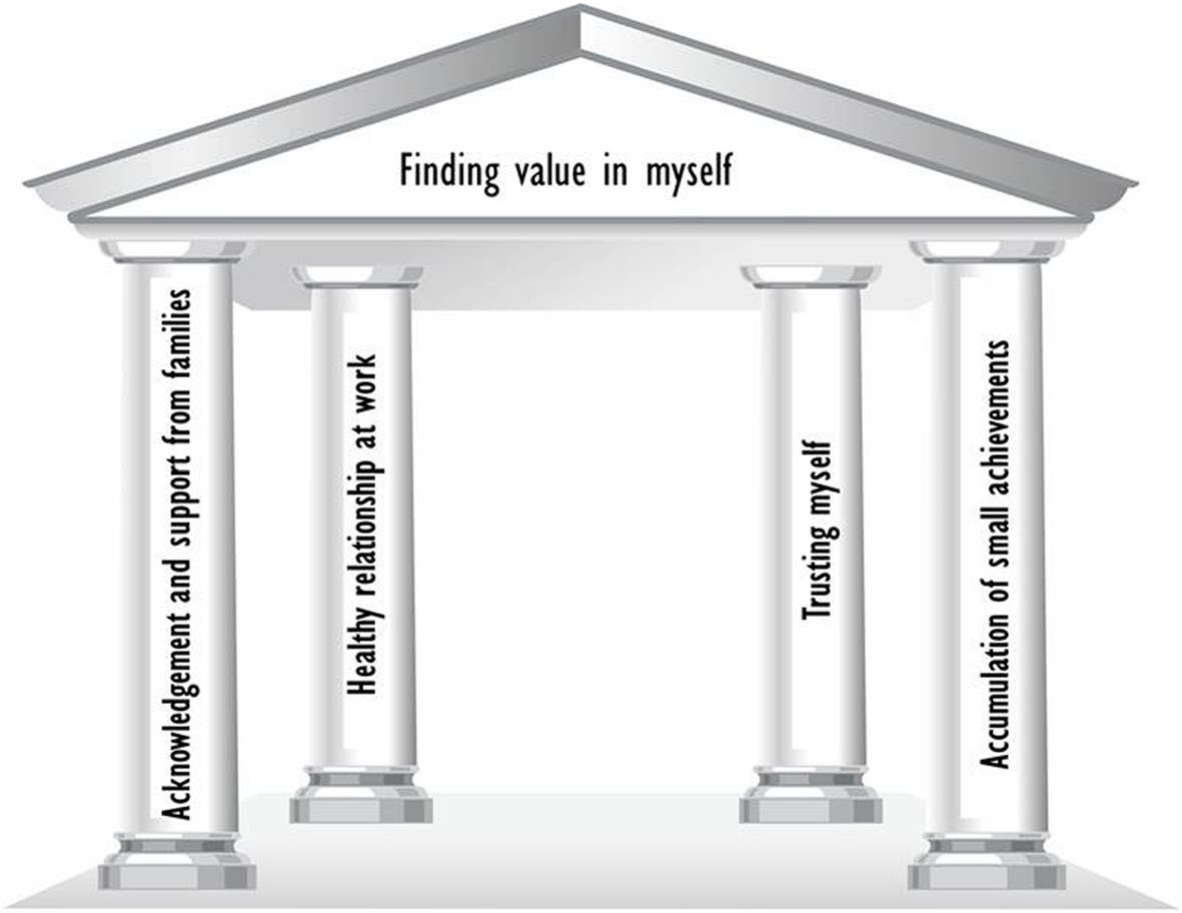
Structure of life as a clinical nurse for nurses with more than 30 years of career retention

To explore the strengths that enabled long-term career retention in the original data of the participants’ narratives, a reanalysis was conducted using Braun & Clarke’s [12] thematic analysis method. The core theme identified “Finding value in myself,” which served as the fundamental strength and helped the nurses achieve a long career of over 30 years. It represents value and joy in the growth of the nurse clinicians, throughout the entire spectrum of their lives. The participants faced difficult moments in which they had to consider changing departments or resigning. However, they regarded these events and situations as opportunities for growth. Their growth could be attributed to receiving acknowledgment and support from their families, their line managers’ support, their trust in themselves, and being valued and appreciated by their patients. This growth was expressed by some participants in terms of endless gratitude for the patient as well as their devoted affection for nursing. It served as a driving force for their career retention. Therefore, merely the adaptation to the events of life turning points encountered in their job is not the entire story. The themes of “acknowledgement and support from family,” “healthy relationships at work,” “trusting myself,” and “accumulation of small achievements,” each served as a pillar of strength. This is consolidated as “Finding value in myself” (Table 2, Fig. 1).

**Table 2:** Analysis of life as a clinical nurse of nurses with more than 30 years of career retention

Category	Theme	Statement
Acknowledgement and support from family	Educational fervor of the parents	ID. All of the four daughters were professionals with a bachelor’s degree (A-01)
		ID. Father’s wish for their daughter’s career to be in nursing (B-02)
		ID. Studying was everything in life (C-02)
	Strong familial love	IT. Childrearing with the participation of all family members (C-37)
		IT. Childrearing with irregularities in time and space settings (C-15)
		IA. Sadness from wandering around for a helping hand in childrearing (D-15)
		IA. My family that I should protect (F-04)
	Proud superwoman	ND. A nurse that family members are proud of (D-31)
		ND. A firm mother based on principles (A-27)
		ND Pride of the children from having a nurse as a mother (A-26)
		ND A sublime and noble image of a mother to her daughter (A-27)
Healthy relationships at work	Infinite strength coming from being acknowledged	NA. Unspoken encouragement from the manager (A-31)
		NA. Leader with reasonable judgment (C-19)
		NA. Work that the manager wants to entrust to my care (E-10)
		NA. Fair and proper support from the line manager (F-03)
	Confronting unreasonable dealings	NA. Evading an emotional relationship (F-01)
		NT. Manager that does not protect the member and sides with the strong (B-22)
		NT. Doctor-friendly manager (E-03)
		NT. Relationship with a doctor that became a source of frustration (F-21)
	Ubiquitous nurse	ND. Health guardian for people around me (F-27)
		ID. Various voluntary activities (B-30)
Trusting myself	Pride in my nursing occupation	NA. A job that can only be done because of me (C-09)
		NA. A job that only I can accomplish (E-10)
	Leader in nursing care with new medical technology	ND. Nursing care with new medical technology starting from my own hand (hemodialysis, hospice care, bone marrow transplantation) (E-11)
		NA Identity as a licensed nurse (D-17)
	Resilient character	IT. Giving up is not acceptable (D-01)
		IA. Character that values faithfulness (A-26)
		IT Self-esteem and pride as an A-class model student (C-04)
		IA. My capability in being able to resolve an urgent situation (A-10)
	Motivating myself	IA. Let us do it properly (B-18)
		IA. Positive attitude toward a target (E-12)
		IA. My place in exchange for a hard struggle (C-08)
		IA. I am not the only one going through this hardship (E-11)
Accumulation of small achievements	Desirable skilled nurse	ND. Pride as a university hospital nurse (F-13)
		ND. Refined and polished along with demonstrating understanding toward humans (F-01)
		ND. Embracing the patient/caregiver who cannot accept the situation (E-18)
		ND. I am weak as a person but strong as a nurse (C-08)
		ND. A nurse that listens to the words instead of trying to teach (E-19)
	Echo of gratitude	ND. Appreciative patients are like my mirror (B-29)
		ND. Dependence on my professional performance (E-10)
		ND. Humble truth of patients asking for help from me (C-31)
		ND. Providing care for the elderly at home (B-29)
	My work that brings me joy and happiness	NA. Workaholic (B-16)
		NT. My work taking priority over childrearing (D-25)
		NA. Rising status of nursing profession (B-20)
		NA. Self-development: master’s degree, Ph.D. (F-03)
		ND. Pride in tenure (A-14)
		ID. Health guardian for people around me (F-27)

Note*.* Preceding the statements, *I* life as an individual, *N* life as nurse, *A* adaptations, *D* dimensions, *T* turnings. In the parentheses after the statements, letters indicate participants and numbers indicate the page of the interview transcript (e.g., A-02 means page 2 of Participant A’s interview transcript)

#### Acknowledgements and support from family

Parents who have supported and guided the study participants in their growth as a nurse helped them cope with stressful situations and provided them with psychological stability. Their parents also enabled them to be healthy.

Their family members have all participated in childrearing, and the presence of strong familial love helps nurses manage both, their work and their family, despite the irregular schedules due to the three-shift rotation at work. Whereas their lives as “superheroes” are challenging, their families take pride in the participants and help them sustain their careers in the nursing field.

*They think “if I ask my mom or my wife, everything is resolved” … not only for health-related problems. All my family members are proud of my working as a nurse. My mother-in-law has kindly taken charge of childrearing and the housework, without which my career would have been impossible to carry out.* (Participant F).

#### Healthy relationships at work

A major motivating factor for them to go to work every day was the relationships established at workplaces; heart-warming comfort and encouragement of demonstrating trust in the participant nurses and acknowledging their competencies in numerous incidents with patients and other medical staff. Negative relationships in the workplace also functioned as a major contributor for participants wanting to leave their careers. However, the positive and healthy relationships they acknowledged were a strong motivating force that kept them in the workplace.

*One of the driving forces that enabled me to stay in the job was and still is the head of the nursing unit... who identifies reasonable points and delivers these points to us …*

*I’ve never had a comfortable rest, whether it was a workday or holiday. I work hard putting all I have into the job but still I receive recognition for that day or holiday.* (Participant A).

#### Trusting myself

This theme, with the most fundamental strength, was inherent in all the participants’ narratives. Although their life paths as nurses were not the ones they had selected, they accepted them as the set path in their lives. Participants continuously encouraged themselves with the mindsets of, “Let us do it properly,” “I can do better,” and “This is a job that only I can do.” These statements were based on their trust and confidence in themselves. In this manner, they have developed themselves as nurse clinicians with a firm authority in their work.

*Ah, today, I performed IVs for 10 new-borns in one go. In the ICU, or in the hematology ward, they look to me for assistance whenever a patient gets worse … that keeps me in the clinical practice … there are rewarding moments … I have set my mind on them.* (Participant E).

#### Accumulation of small achievements

This theme reveals that when nurses have job satisfaction and are appreciated in the field of nursing care, they gain the motivation to retain their career as nurse clinicians. In addition to a range of excellence awards, continuous-service awards, and financial rewards, nurses value the patients they encounter even more. Their internal maturity is another aspect of pride and joy that only the nurses themselves appreciate. Numerous patients expressing gratitude for their words and their nursing activities are perceived as a great attraction for the participants, encouraging them to remain in their careers. Another positive motive is patient appreciation, for example, preventing an emergency or more serious problem.

*I am only a humble nurse but the patients keep coming to me, saying, “Please help me with this” or “Thank you.” In that moment, I realized that they are all valuable people of whom I should be appreciative. Nursing truly is a profession that has transformed me from a person who had a narrow, self-centered perspective to a person with a more enriched understanding of human beings.* (Participant F).

## Discussion

First, we would like to compare the results of Mandelbaum’s life history narrative analysis, which is distinguished by dimensions, turnings, and adaptations, with the lives of contemporary Korean women.

The study focuses on the lives of those nurse clinicians in the baby boomer generation, whose careers began in the early and mid-1980s and continue to date, spanning over 30 years. These nurses’ career trajectories reveal a close connection with and relationship between the changes in the social aspects, the fields of healthcare and nursing, their individual predispositions, family recognition, and organizational support. This was the generation in which the societal atmosphere allowed women to have a professional career. It was also the first generation in which the social status of nurses was elevated, such that they now work with recognition from home as well as at work. In Korea, career retention for women was made possible after marriage, from the mid-1980s. From then on, there were various changes regarding women’s educational level, age of recruitment, age of retirement, and type of occupation [14]. In this study, the social image of Korea was clearly divided into two dimensions: “individual” as a natural person and “nurse” as a profession.

Since the 1990s, the number of women who have professional careers and participate in social activities has increased, and the concept of “career women” has developed [15]. The study’s findings reveal that participants’ family members including their parents, siblings, spouses, and children have referred to the participants as superheroes. This indicates a sense of pride and an acknowledgement of their loved one as a nurse, reflecting the same trend and context as mentioned previously. However, Korean society expected women to be helpers and responsible for their children’s upbringing, within their families. Korean society took women’s sacrifice, both personal and professional, and dedication to their families for granted [15]. Although this study’s participants had different lifestyles, they were all bound by their responsibility as mothers and homemakers, where considerable input and personal resources were channeled into child-rearing. Child-rearing stress in nurses is higher than that in professionals in other fields, due to the former’s irregular working conditions, work shift patterns, and the added burdens or expectations they experience at home [16].

Marriage and child-rearing were positioned as the most important “turning” events in a woman’s life in this era. As women’s educational levels increased, their perception of lifelong employment also improved. However, child-rearing and care work were still recognized primarily as a woman’s role. Therefore, the children of a dual income family were regarded as “those who were deficient in some respects.” Consequently, working mothers had to “feel sorry for their children.” [17] Although the participants worked hard in both their professional and private lives, they felt some guilt when reflecting on how they had to approach their childcare responsibilities.

For an individual, a family is the most important and closely connected social environment. The functions performed by a family affect the psychological and social characteristics of family members and the unit as a whole [18]. Additionally, family support is instrumental in providing a safe haven for rest and recovery, and is a factor that reduces work-family conflict [19]. Regarding child-rearing, in particular, spousal support lowers parental stress [20]. The study participants were able to solve the problem of maintaining a work-family balance because of their families’ (including their husbands’) sacrifice and support. In the narratives, the participants mentioned that their families’ understanding, support, and encouragement were some of the major strengths that helped them keep their jobs. This outlook arose from a comparison with their older colleagues, who were forced to abandon their nursing careers after marriage.

An important “turning” event in their lives as nurses was their interpersonal relationships at work. For these participants, interpersonal relationships at work posed a threat to their job retention. In some circumstances, however, these relationships also acted as a strength that allowed them to maintain their careers. Social support at work included support from managers and colleagues [21]. This study’s results reveal that the participants retained their careers because they had respect and positive interpersonal relationships with their supervisors and nurse managers. These findings are supported by that of an earlier study, where nursing managers’ abilities and support were predictors of nurses’ intention of career retention. This had a positive effect on the relationships with their nursing colleagues [22].

In line with the results of a prior study, participants in this research overcame difficult moments, came even close to resignation, with the support of the nursing unit members; it shows that sharing job-related information among their colleagues within the organization and having emotional support, such as psychological comfort and interest, contributes to job retention [23]. This study’s results also reflect the same context as that of another [24], which reported that amicable cooperation with doctors serves as a motivator and plays an important role in preventing nurse turnover intention. Further, this study’s results are also consistent with a previous finding: for female teachers, having no colleague to perform a given task in collaboration with, or having no senior member as a role model or mentor, serves as a great obstacle to their career retention [25].

“Adaptations” of life history should be discussed together with the results of the narrative analysis on the strength of long-term career retention of more than 30 years. Where did the strength of working as a clinical nurse for more than 30 years come from? That strength was extended to “adaptation” because of interpreting and living through the “turning” events revealed in the life story narratives of the nurses. Since having a job is an indispensable determinant for the quality life for modern people, the value placed on it are linked to the overall perspective on life, and life values [14]. By examining the participants’ expressions, “having a calling” and their work being perceived as a “vocation,” we can understand their professional perspectives. Placing significance on their profession’s value to society and themselves enabled the participants to retain their careers over a long duration.

The greatest significance of “work” lies in its role as a means of providing a livelihood for the family. However, “work” and “workplace” have different meanings in various dimensions. Here, work includes both aspects of values and stress in life [26]. Nurses feel rewarded and develop a high self-esteem through patient care. As a nurse, their role is acknowledged by their patients and their patients’ caregivers as meaningful, when they perform their responsibilities well [27]. Autonomy in work, where nurses take the initiative, serves as a positive motivator [28]. It allows them to make their own decisions and to solve problems, which affect their job retention [23]. This is consistent with the motivational factors in Herzberg’s [29] motivation-hygiene theory, in which an individual seeks to realize one’s potential through continuous mental growth. This growth and development encouraged the present study’s participants to reinforce their concept of nursing as nurse clinicians and contributed positively toward their pride and self-esteem.

Nursing professionals’ values reflect their beliefs, ideas, and impressions about nursing and about nursing as a profession [30]. The concept encompasses all aspects of their systematic opinions on nursing, the process of nursing activities, and their occupational opinions on the profession [31]. Nurses’ professional values are influenced by socialization factors; individual thoughts and beliefs are formed and act as the basis for their nursing behavior [31]. This affects the establishment of a nurse’s identity [32], and provides a sense of job satisfaction as well as social recognition in the value of nursing care [33].

This study’s participants were working as new graduate nurses during the 1980s, when the change in the status of women and nurses in the Korean society led to significant changes in the implications of living as a professional and a nurse. This also applied to their status in the field of healthcare and the nursing community. These aspects positively contributed to their career retention. The participants’ faithfulness and sense of responsibility are revealed in their experiences and via the process of exploring the inherent meaning of nursing. It should be noted that individual values play an important role in a clinical nurse’s career retention. Further, this study’s finding that the participants’ strengths assisted them in retaining their long-term careers over 30 years, is consistent with a previous study’s results [33]. This prior study reported that the higher the nursing professional’s values, the higher their intentions for career retention.

From the expressions used by this study’s participants, referring to their job as their “path,” or a “calling, indeed,” it can be observed that their profession’s meaning includes the moral implication that their work is performed as a calling. This is in addition to the meaning of doing labor for a living. This implication reflects the same context as a previous study’s finding [25] that assigning positive meanings to a job forms the basis for job commitment and occupational orientation. The participants continued their service as nurse clinicians while fulfilling their responsibilities as the pioneering leaders of clinical care and nursing. This changed over time, in line with the development of each period.

Since this study tells the story of clinical nurses who have lived in a specific era in a specific society, it is limited in that it may not be applicable to other societies, different clinical settings, and nurses’ life history narratives in other eras. However, this limitation is characteristic of qualitative research.

## Conclusion and suggestions

This study was initiated with the question of identifying nurse clinicians’ essential strength that enabled them to retain their careers for a period of over 30 years, by using their life-history narratives. We reviewed their values from various perspectives through the overall flow in their lives and the specific phenomena or actions they underwent during their lifetimes.

The core strength that allowed the participants to maintain long-term careers was “Finding value in myself” through their work, representing the values and joy acquired in their overall lives regarding their own growth as clinical nurses. Various dimensions in their lives did not collapse, but formed a balance. Each dimension served as a lever for the other. Thus, the driving force of “Finding value in myself” was maintained.

Based on this study’s results, we present the following suggestions. For the retention of nurses’ long-term service, on the clinical dimension: creating a culture of positive and mutually respectful interpersonal relationships within the organization is instrumental. Additionally, adjusting the job-intensity and their workload as well as increasing hands-on nursing services are expected to enhance job satisfaction. This is obtained through positive feedback from the patients. On the individual dimension, the experience of healthy interpersonal relationships and efforts to promote their professional values are required. Since the individual nurse’s professional values reflect the era’s social and cultural values, a further investigation into the life history of nurses from post-baby boomer generation and various socio-cultural backgrounds is advised.
